# Incidence and independent risk factors for postoperative delirium in ICU patients after cardiopulmonary bypass cardiac surgery: a retrospective cohort study

**DOI:** 10.3389/fcvm.2026.1868118

**Published:** 2026-07-06

**Authors:** Chunyan Zhang, Huan Yu, Wei Zhai, Lili Zhang, Qing Li, Yuanyuan Li

**Affiliations:** Department of Cardiovascular Surgery, The Second Hospital of Tianjin Medical University, Tianjin, China

**Keywords:** extracorporeal circulation cardiac surgery, ICU nursing records, incidence, influencing factors, postoperative delirium

## Abstract

**Objective:**

This study aims to investigate the incidence of delirium in patients admitted to the intensive care unit (ICU) after extracorporeal circulation cardiac surgery and analyze the key influencing factors.

**Method:**

A retrospective cohort study was conducted. 302 patients who underwent extracorporeal circulation cardiac surgery and were transferred to the ICU in our hospital from January 2023 to June 2025 were continuously included. According to the ICDSC results in the ICU nursing records, the patients were divided into the delirium group (POD, *n* = 85) and the non-delirium group (Non-POD, *n* = 217). Baseline data, perioperative and early postoperative indicators were compared. Multivariate logistic regression identified independent risk factors, and SHAP, ROC curves were analyzed.

**Result:**

The incidence of POD was 28.15%. Compared with the Non-POD group, the POD group had significantly higher age, APACHE II score, sedative use, postoperative hypotension rate, and lower albumin level, as well as longer aortic cross-clamping time, cardiopulmonary bypass time, and mechanical ventilation time (all *P* < 0.05). Multivariate analysis showed that older age, longer mechanical ventilation time, longer aortic cross-clamping time, higher APACHE II score, and lower albumin levels were independent risk factors (AUC: 0.719, 0.769, 0.677, 0.782, and 0.845, respectively, Mean |SHAP|: 1.58, 1.96, 1.01, 0.95, 2.81).

**Conclusion:**

The incidence of POD after extracorporeal circulation cardiac surgery is relatively high. Its occurrence is closely related to advanced age, aortic cross-clamping time, postoperative mechanical ventilation time, APACHE II score, and albumin content. ICU nursing records can effectively identify high-risk patients. The research results provide key evidence for early warning, risk stratification, and the formulation of targeted prevention and intervention strategies in clinical nursing practice.

## 1 Introduction

Extracorporeal circulation is often used in 70% of cardiac surgeries, and cardiac surgery with extracorporeal circulation is the core technology in modern cardiothoracic surgery for treating complex cardiac structural disorders and coronary artery diseases (1). It provides surgeons with a clear and blood-free surgical field and ample operation time by temporarily replacing the functions of the heart and lungs, thus enabling them to successfully perform a series of highly challenging surgeries (2). However, this life-supporting technology, while creating the surgical conditions, also brings a series of systemic pathological and physiological challenges (3). This surgery may cause surgical trauma stress, extracorporeal circulation inflammation, impaired cerebral blood flow regulation and microembolism formation, which will inflict multiple blows to the patient's central nervous system (4). In this context, postoperative delirium (POD), as an acute brain dysfunction, has become one of the most common central nervous system complications following cardiac surgery with extracorporeal circulation (5). POD is not merely a “state of confusion of consciousness”. Its core feature lies in the acute onset of attention disorders and changes in consciousness level, accompanied by fluctuating disorders in cognitive functions or perception (6). The pathophysiological mechanism of POD is complex, involving the activation of neuroinflammation, imbalance of neurotransmitters, disruption of the circadian rhythm, and various networks related to brain energy metabolism crisis (6). A large amount of evidence-based medical data indicates that among patients undergoing cardiac surgery with extracorporeal circulation, the overall incidence of POD can be as high as approximately 30% (5, 7, 8). There is evidence suggesting that POD is an independent risk factor that accelerates long-term cognitive decline in patients, increases the risk of dementia, and leads to long-term functional dependence and a decline in quality of life, posing a significant threat to patients' rehabilitation and quality of life (9).

The Intensive Care Unit (ICU) is the core location where post-heart surgery patients receive life support and organ function recovery. However, the ICU environment itself may also contribute to the occurrence and development of delirium (10). The ICU environment is complex and unique. Continuous alarms, irregular lighting, and frequent nursing procedures result in severe fragmentation of patients’ sleep or even deprivation of sleep (11). In addition, the treatment itself also poses risks. Deep sedation may induce or mask POD. Multiple analgesic and sedative drugs are prone to produce synergistic or cumulative effects. Mechanical ventilation causes discomfort and communication barriers, and restraints exacerbate anxiety and helplessness (12). In this complex context, the value of ICU nursing records has gained recognition. As an objective, continuous and dynamic document carrier for clinical frontline nursing work, the modern structured electronic nursing record system is no longer merely a simple description of the patient's condition; instead, it is a collection of data integrated with standardized assessment tools (13). These structured and sequential data provide researchers with valuable and reliable data sources for retrospective analysis, making it possible to accurately identify the onset time of delirium, calculate the incidence rate in the real world, analyze its diurnal fluctuation patterns, and explore its potential correlations with various clinical indicators (14). However, although the clinical significance of POD has been widely recognized, at present, in the specific medical environment of our country, with full utilization of these high-frequency and structured ICU nursing record data, and specifically targeting the high-risk group of extracorporeal circulation heart surgery, there is still a lack of comprehensive and in-depth retrospective studies on multiple influencing factors with large sample sizes. This limitation restricts the construction of a precise risk profile for this group and the optimization of local prevention strategies.

Although traditional Logistic regression can identify the independent risk factors for POD and provide odds ratios, it has limitations in revealing nonlinear relationships, interactions, and individual-level risk attribution among variables (15). Moreover, relying solely on discriminative indicators such as AUC is insufficient to evaluate the actual benefits of the prediction model in real clinical decision-making (16). In recent years, the SHAP (Shapley Additive Explanations) method can achieve model interpretability and individualized risk analysis by decomposing the marginal contribution of each feature to the individual prediction results (17); Decision Curve Analysis (DCA) can quantify the clinical net benefits of the model under different risk thresholds, compensating for the shortcomings of traditional assessment indicators (18). Currently, in the research on the prediction of POD after extracorporeal circulation cardiac surgery, there are no reports of jointly applying Logistic regression, SHAP, and DCA, and systematically evaluating the performance and clinical utility of the model. Therefore, in this study, based on the construction of the Logistic regression model, the SHAP method is further introduced to analyze the contribution of individual-level risk factors, and DCA is used to evaluate the clinical applicability of the model, in order to provide more sufficient methodological basis for the early identification of high-risk patients and the optimization of nursing intervention strategies.

In conclusion, although several risk factors have been identified, including advanced age, prolonged aortic occlusion time during surgery, and postoperative sedation, the existing evidence mostly comes from small sample, single-center studies, and mostly employs traditional regression analysis methods. This makes it difficult to reveal the nonlinear relationships between variables and the individual-level risk attribution. Moreover, there is currently a lack of predictive model research based on structured ICU nursing records, combined with interpretable machine learning methods and clinical decision utility assessment. Therefore, this study is not a simple local data validation. Instead, it utilizes high-quality structured nursing record data to jointly apply Logistic regression, SHAP values, and decision curve analysis in the post-cardiac surgery population with cardiopulmonary bypass. It systematically evaluates the individualized contributions of each risk factor and the clinical net benefit of the model, with the aim of compensating for the deficiencies in the model's interpretability and clinical practicality evaluation in existing literature.

## 2 Research subjects and methods

### 2.1 Research subjects

**Figure 1:**
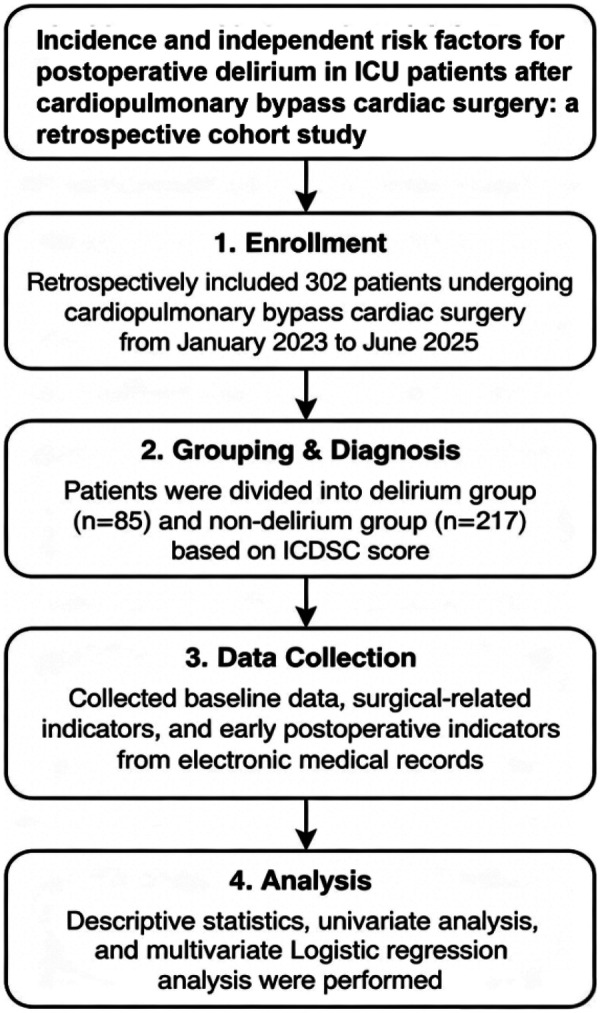
Study flow diagram of patient selection. POD, postoperative delirium; ICDSC, intensive care delirium screening checklist; ICU, intensive care unit.

This study is a single-center, retrospective, observational cohort study. A total of 302 patients who underwent extracorporeal circulation heart surgery (such as coronary artery bypass grafting, heart valve surgery) in the cardiac surgery department of our hospital and were directly transferred to the cardiac surgery ICU from January 2023 to June 2025 were consecutively included. According to the ICDSC results in the ICU nursing records, the patients were divided into the delirium group (*n* = 85) and the non-delirium group (*n* = 217). This study was approved by the The Second Hospital of Tianjin Medical University's ethics committee (approval number: KY2026K208). Given that it is a retrospective study, patient informed consent was waived, but all patient data were anonymized and kept confidential. The patient selection process is illustrated in Figure 1.

Inclusion criteria: (1) Age≥18 years; (2) Patients undergoing cardiac surgery under cardiopulmonary bypass; (3) Patients admitted to the ICU for postoperative treatment; (4) Patients with complete and usable electronic nursing records in the ICU.

Exclusion criteria: (1) Patients with severe cognitive impairment, dementia, or history of mental illness; (2) Patients who were in a coma or had severe neurological diseases before the surgery [Severe complications are defined as events with a Clavien-Dindo classification of ≥grade IV (19).]; (3) Patients who abandoned treatment early due to non-medical reasons or died/ were transferred out within 24 h after the surgery; (4) Patients with incomplete or missing assessment records of delirium in the ICU nursing records; (5) Patients who were in a coma or had severe complications after the surgery.

### 2.2 Diagnostic and scoring criteria

The patients were grouped based on whether they had positive records of POD that met the diagnostic criteria in their ICU care records. The diagnosis of POD was based on the records in the ICU care files, which were evaluated using standardized tools “Intensive Care Delirium Screening Checklist” (ICDSC) on a daily basis as per the established procedures, an ICDSC score of 4 or higher indicates the presence of delirium; a score of 1 to 3 indicates mild delirium (subsyndromal delirium) (20, 21). This assessment is conducted and recorded by trained nurses in the intensive care unit. At least once during the assessment, patients with an ICDSC score of 4 or higher (the delirium threshold) are classified into the POD group, while patients with a score consistently below 4 throughout the intensive care period are classified into the non-POD group.

NYHA classification (22) is a subjective grading based on the degree of physical activity limitation and heart failure-related symptoms (such as shortness of breath, palpitations, fatigue) in patients. Grade I indicates no limitation in activity and no symptoms during daily activities; Grade II indicates mild limitation in activity and symptoms occur during daily activities; Grade III indicates significant limitation in activity and symptoms occur at activities below daily level; Grade IV indicates inability to move and symptoms occur even at rest. This classification is determined by experienced clinicians with 10 years of experience after collecting the medical history, and it needs to be clearly stated in the medical record. The assessment is conducted within 24 h before the operation.

ASA classification (23) classifies the physical condition of patients into six grades based on the preoperative comorbid systemic diseases and their interference with physiological functions: Grade I (normal health), Grade II (mild systemic diseases), Grade III (severe systemic diseases), Grade IV (life-threatening severe diseases), Grade V (near death and unable to survive without surgery), Grade VI (brain death organ donation). Emergency surgeries require an “E” suffix after the classification. The assessment is completed by an experienced anesthesiologist within 24 h before the operation.

APACHE II score (24) consists of three parts: Acute Physiology Score (APS) collects the worst 12 physiological indicators within 24 h after admission to the ICU (each item 0–4 points); Age score (0 points for≤44 years old and 6 points for ≥75 years old); Chronic Health Status Score (5 points for patients with a history of severe organ dysfunction and emergency or non-surgical patients, 2 points for elective surgery patients, and 0 points for those without). The total score is the sum of the three (range 0–71 points), and the higher the score, the more severe the condition and the higher the in-hospital mortality rate. This score is conducted by experienced physicians with 10 years of experience. The assessment should be conducted within 24 to 48 h after the patient is admitted.

### 2.3 Data collection

Baseline data of the two groups of patients were collected from the hospital electronic medical record system and the ICU nursing record system (gender, age, body mass index, education level, smoking history, drinking history, chronic diseases (hypertension, diabetes, hyperlipidemia), cardiovascular surgery history, NYHA cardiac function classification, ASA classification, APACHE II score, surgery type (simple valve surgery or coronary artery bypass grafting or other surgeries, mixed surgeries or aortic-related surgeries), operation duration, blood loss, aortic occlusion duration, intraoperative sedation, analgesic drug use, extracorporeal circulation time and early postoperative indicators (postoperative hypotension: Within 24 h after the operation, if the MAP was less than 65 mmHg or the SBP was less than 90 mmHg for a duration of ≥10 min, or if vasopressor drugs were needed to maintain blood pressure. If a patient experienced at least one episode of hypotension meeting the above criteria, they were included in the postoperative hypotension positive group (25), mechanical ventilation time, ICU stay time, total hospital stay time, hemoglobin, albumin level).

### 2.4 Statistical analysis

Data analysis was conducted using SPSS 27.0 software (IBM Corporation, Armonk, NY, USA). Normality and homogeneity of variance tests were performed for all variables. For continuous variables that followed a normal distribution and had homogeneity of variance, the mean ± standard deviation ($\bar{x}\pm s$ was used for description, and *t*-tests were employed for comparisons between the two groups; for non-normal distribution of measurement data, the median and interquartile range were used for representation, and the Wilcoxon test was applied for comparisons between groups; for categorical data, the number of cases (percentage) was used, and the *χ*^2^ test was employed for comparisons between groups. Variables with *P* < 0.05 in the univariate analysis were included in the multivariate Logistic regression model (forward stepwise method) to identify independent risk factors for POD, and the odds ratio (OR) and its 95% confidence interval (CI) were calculated. All tests were two-sided, and a *P* < 0.05 was considered statistically significant.

## 3 Results

### 3.1 Comparison of general information

The incidence of POD was 28.15%. The age of POD patients was higher than that of Non-POD patients (*P* < 0.05), while there were no differences in gender, BMI, education level, and previous medical history (*P* > 0.05). See Table 1.

**Table 1:** Comparison of general information.

Index	Non-POD (*n* = 217)	POD (*n* = 85)	*t/χ*²	*P*
Gender/*n* (%)			0.137	0.711
Male	120 (55.30)	45 (52.94)		
Female	97 (44.70)	40 (47.06)		
Age (year, $\bar{x}\pm s$)	60.31 ± 7.38	66.19 ± 7.13	6.530	<0.001
BMI (kg/m², $\bar{x}\pm s$)	22.74 ± 3.96	22.82 ± 3.50	−0.175	0.861
Highest education attained/*n* (%)			0.133	0.936
Primary school or below	40 (18.43)	17 (20.00)		
Junior or senior high school	107 (49.31)	42 (49.41)		
College or bachelor's degree or above	70 (32.26)	26 (30.59)		
Smoking history/*n* (%)			0.458	0.499
Yes	62 (28.57)	21 (24.71)		
No	155 (71.43)	64 (75.29)		
Alcohol consumption history/*n* (%)			1.126	0.289
Yes	67 (30.88)	21 (28.24)		
No	150 (69.12)	64 (71.76)		
Hypertension/*n* (%)			0.003	0.957
Yes	44 (20.28)	17 (20.00)		
No	173 (79.72)	68 (80.00)		
Diabetes/*n* (%)			0.032	0.858
Yes	35 (16.13)	13 (15.29)		
No	182 (83.87)	72 (84.71)		
Hyperlipidemia			0.026	0.872
Yes	27 (12.44)	10 (11.76)		
No	190 (87.56)	75 (88.24)		

POD, postoperative delirium; BMI, body mass index; SD, standard deviation. All continuous variables were tested for normality using the Kolmogorov–Smirnov test before analysis, and chi-square test was used for categorical variables. Independent sample t-test was used for continuous variables. *P* < 0.05 indicated statistical significance.

### 3.2 Comparison of clinical data

The POD group had higher age, APACHE Ⅱ score, postoperative sedation, postoperative hypotension rate, and lower albumin level (*P* < 0.05), and longer aortic occlusion time, cardiopulmonary bypass time, and mechanical ventilation time compared to the Non-POD group (*P* < 0.05). There were no differences in other parameters such as operation duration, operation type, blood loss, NYHA and ASA scores, ICU stay time, total hospital stay, and hemoglobin (*P* > 0.05). See Table 2.

**Table 2:** Comparison of clinical data.

Index	Non-POD (*n* = 217)	POD (*n* = 85)	*t/χ*²	*P*
History of cardiovascular surgery/*n* (%)			0.085	0.770
Yes	23 (10.60)	10 (11.76)		
No	194 (89.40)	75 (88.24)		
NYHA classification/*n* (%)			0.331	0.565
Ⅰ–Ⅱ	66 (30.41)	23 (27.06)		
Ⅲ–Ⅳ	151 (69.59)	62 (72.94)		
ASA classification/*n* (%)			0.535	0.465
Ⅰ–Ⅱ	135 (62.21)	49 (57.65)		
Ⅲ	82 (37.79)	36 (42.35)		
Type of surgery/*n* (%)			0.241	0.623
Single type	174 (80.18)	66 (77.65)		
Mixed type	43 (19.82)	19 (22.35)		
Duration of the surgery (h, $\bar{x}\pm s$)	3.38 ± 0.55	3.40 ± 0.66	−0.213	0.832
Amount of bleeding (mL, $\bar{x}\pm s$)	225.29 ± 23.71	226.13 ± 22.22	−0.282	0.778
Aortic occlusion time (min, $\bar{x}\pm s$)	59.19 ± 6.23	63.36 ± 6.03	−5.258	<0.001
Cardiopulmonary bypass time (min, $\bar{x}\pm s$)	81.80 ± 8.65	84.95 ± 7.93	−2.904	0.004
Mechanical ventilation duration (h, $\bar{x}\pm s$)	20.23 ± 3.07	23.33 ± 3.00	−7.908	<0.001
APACHE II score (points, $\bar{x}\pm s$)	10.08 ± 1.93	12.05 ± 1.50	8.236	<0.001
Postoperative sedation/n (%)			10.679	0.001
Yes	90 (41.47)	53 (62.35)		
No	127 (58.53)	32 (37.65)		
Postoperative analgesia/n (%)			0.185	0.667
Yes	174 (80.18)	70 (82.35)		
No	43 (19.82)	15 (17.65)		
Postoperative hypotension/n (%)			12.206	<0.001
Yes	113 (52.07)	63 (74.12)		
No	104 (47.93)	22 (25.88)		
Length of stay in the ICU (d, $\bar{x}\pm s$)	5.00 ± 0.99	5.14 ± 0.92	−1.118	0.265
Total length of hospital stay (d, $\bar{x}\pm s$)	17.98 ± 2.16	18.13 ± 2.07	−0.560	0.576
Hemoglobin (g/L, $\bar{x}\pm s$)	134.76 ± 14.24	133.32 ± 14.51	0.782	0.435
Albumin (g/L, $\bar{x}\pm s$)	42.22 ± 4.23	36.09 ± 4.37	11.163	<0.001

POD, postoperative delirium; NYHA, New York heart association; ASA, American society of anesthesiologists; APACHE II, acute physiology and chronic health evaluation II; ICU, intensive care unit; SD, standard deviation. Chi-square test was used for categorical variables. For continuous variables, independent sample *t*-test was employed after confirming normality and homogeneity of variance. *P* < 0.05 indicates statistical significance.

### 3.3 Logistic regression analysis

**Figure 2:**
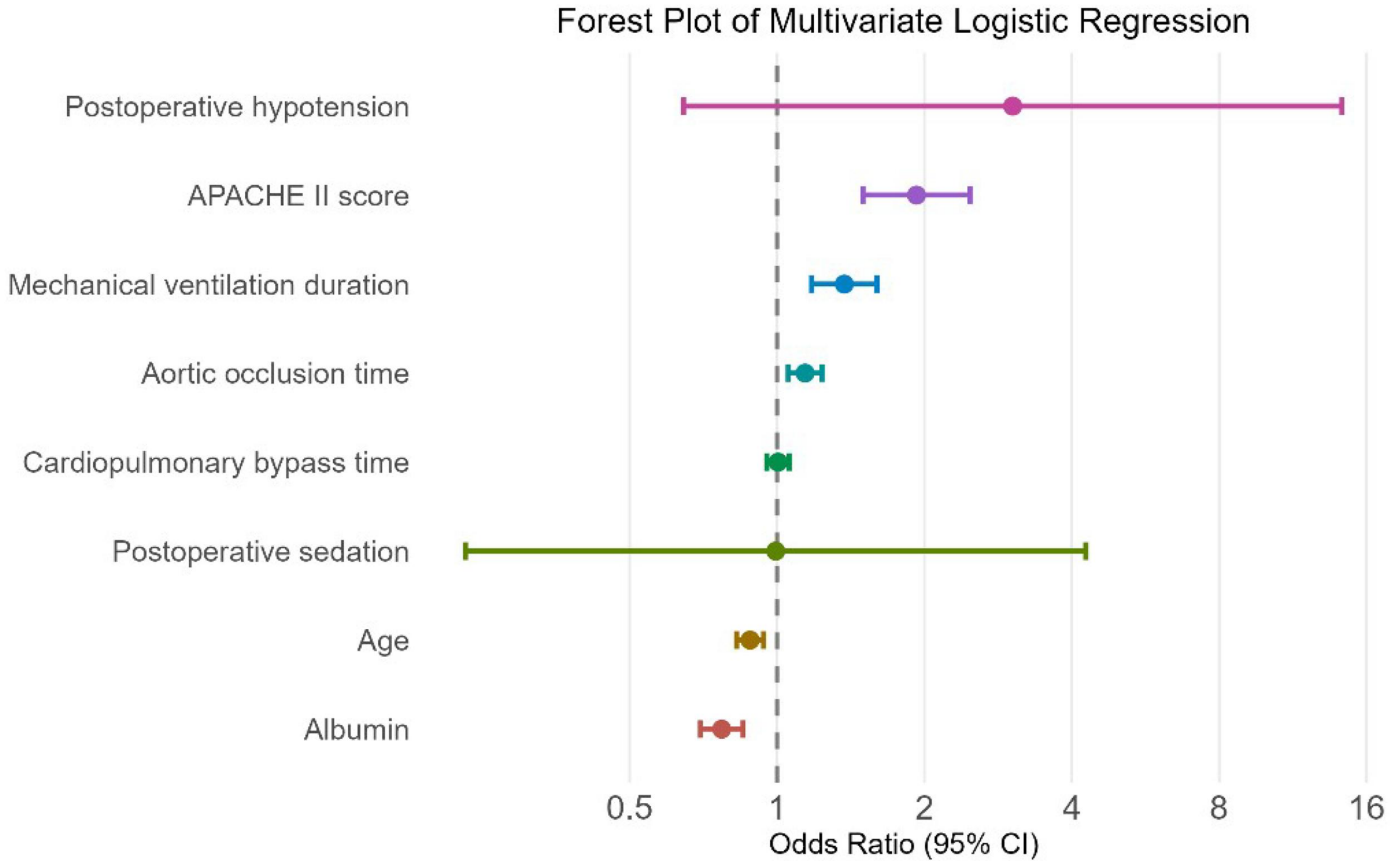
Forest plot of multivariate logistic regression analysis for risk factors of POD. POD, postoperative delirium; OR, odds ratio; CI, confidence interval; APACHE II, Acute Physiology and Chronic Health Evaluation II. The dependent variable was the occurrence of POD (1 = POD, 0 = non-POD). Each square represents the point estimate of the OR, and the horizontal error bars indicate the 95% CI. The vertical dashed line at OR = 1 represents the line of no effect; variables with OR > 1 (to the right of the reference line) are risk factors, while variables with OR < 1 (to the left) are protective factors.

The multivariate analysis revealed that older age (OR, 1.134; 95% CI: 1.065–1.207; *P* < 0.05), longer aortic occlusion time (OR, 1.141; 95% CI, 1.054–1.236; *P* < 0.05), longer mechanical ventilation time (OR, 1.372; 95% CI, 1.177–1.599; *P* < 0.05), higher APACHE II score (OR, 1.927; 95% CI, 1.499–2.477; *P* < 0.05), and lower albumin levels (OR, 0.771; 95% CI, 0.698–0.852; *P* < 0.05) were independent risk factors for POD. See Table 3 and Figure 2.

**Table 3:** Logistic regression analysis of the occurrence of POD.

Index	*β*	S.E.	Wald *χ*²	*P*	OR	95% CI
Age	0.126	0.032	15.210	<0.001	1.134	1.065–1.207
Mechanical ventilation duration	0.316	0.078	16.361	<0.001	1.372	1.177–1.599
Cardiopulmonary bypass time	0.005	0.027	0.036	0.850	1.005	0.953–1.060
Aortic occlusion time	0.132	0.041	10.561	0.001	1.141	1.054–1.236
APACHE II score	0.656	0.128	26.213	<0.001	1.927	1.499–2.477
Postoperative sedation	−0.006	0.744	0.000	0.993	0.994	0.231–4.274
Postoperative hypotension	1.108	0.790	1.967	0.161	3.029	0.644–14.252
Albumin	−0.260	0.051	26.093	<0.001	0.771	0.698–0.852

*β*, regression coefficient; S.E., standard error; Wald *χ*^2^, Wald chi-square statistic; OR, odds ratio; CI, confidence interval; APACHE II, acute physiology and chronic health evaluation II; The dependent variable is the occurrence of postoperative delirium (POD) (1 = has POD, 0 = has no POD). The upper and lower limits of the 95% CI values are indicated. *P* < 0.05 indicates statistical significance.

### 3.4 Contributiveness and ROC analysis

**Figure 3:**
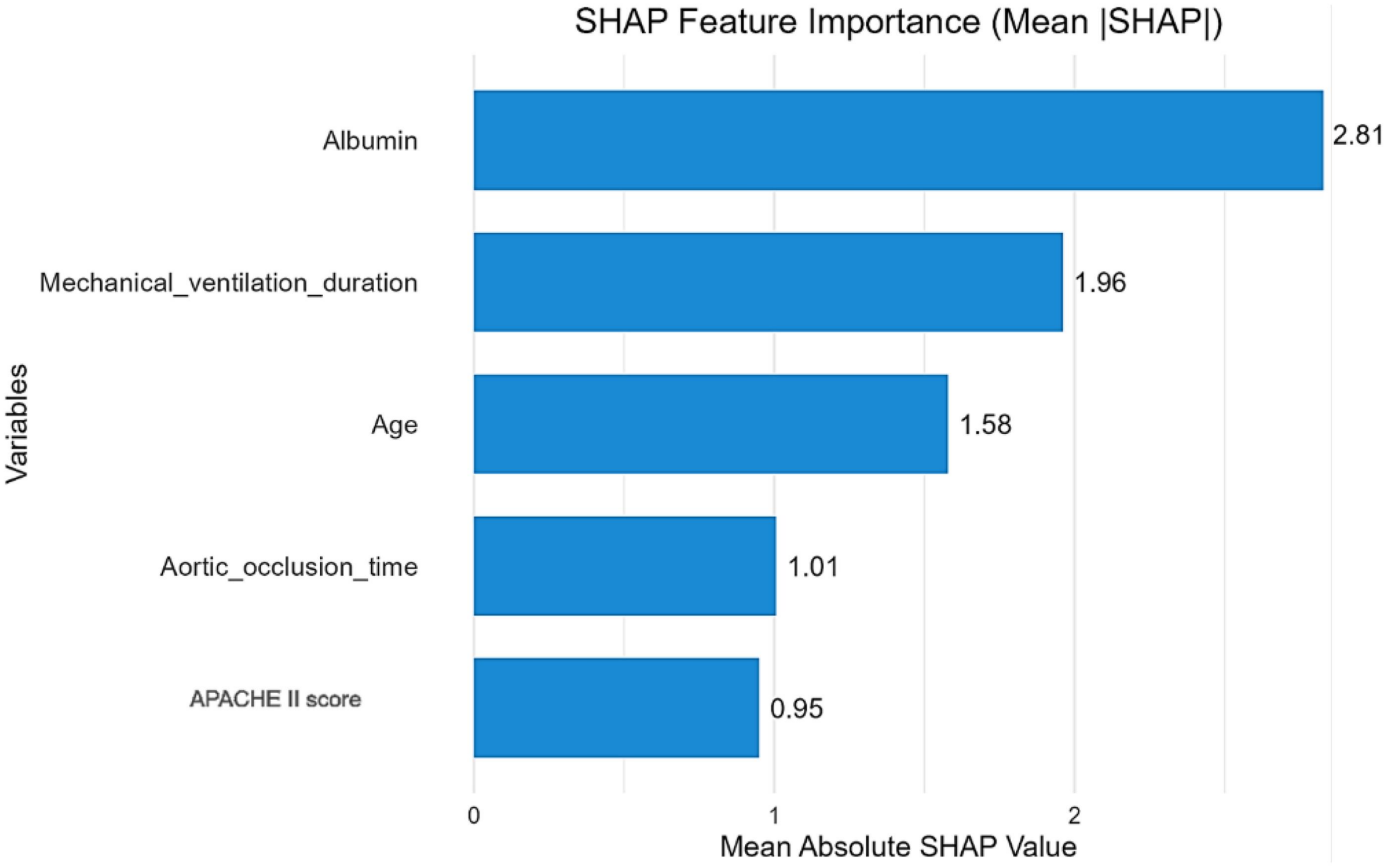
SHAP summary plot of feature importance for predicting POD. SHAP, shapley additive explanations; POD, postoperative delirium; APACHE II, acute physiology and chronic health evaluation II. This figure presents the mean absolute SHAP values for each predictor variable in the logistic regression model. The bars represent the mean absolute SHAP values across all patients; longer bars indicate greater overall contribution to the predictive model.

The SHAP analysis revealed that the average absolute SHAP value of Albumin was the highest, at 2.81; while APACHE II score had the lowest value, at 0.95. See Figure 3.

**Figure 4:**
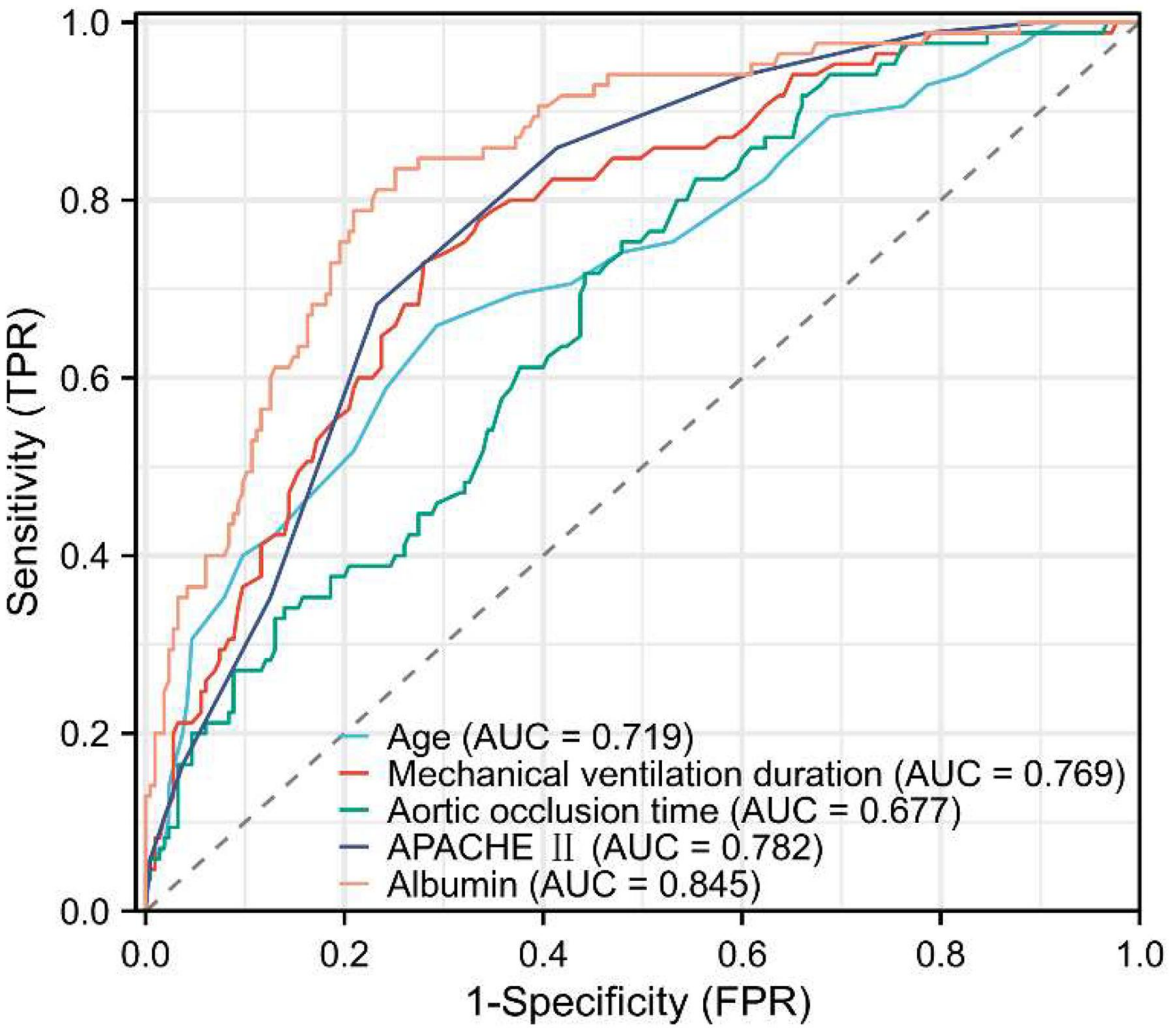
ROC curves of individual predictors and the combined model for POD. POD, postoperative delirium; ROC, receiver operating characteristic; AUC, area under the curve; APACHE II, acute physiology and chronic health evaluation II. The ROC curves plot sensitivity against 1-specificity for each predictor. The AUC quantifies the discriminative ability of each predictor, with values ranging from 0.5 (no discrimination) to 1.0 (perfect discrimination).

The AUC values for the five risk factors-Age, Mechanical ventilation duration, Aortic occlusion time, APACHE II score, and Albumin-were 0.719, 0.769, 0.677, 0.782, and 0.845 respectively. The Sensitivity values were 0.707, 0.512, 0.628, 0.414, and 0.749 respectively. The Specificity values were 0.659, 0.141, 0.129, 0.141, and 0.835 respectively. In addition, the AUC of the combined model was 0.876, the sensitivity was 85.2%, the specificity was 82.3%, and the Youden index was 0.675. See Table 4 and Figure 4.

**Table 4:** ROC analysis of the occurrence of POD.

Index	AUC	Cutoff value	Sensitivity	Specificity	*J*
Age	0.719	62.500	0.707	0.659	0.366
Mechanical ventilation duration	0.769	20.150	0.512	0.141	−0.347
Aortic occlusion time	0.677	56.950	0.628	0.129	−0.243
APACHE II score	0.782	10.500	0.414	0.141	−0.445
Albumin	0.845	39.745	0.749	0.835	0.584
Combined model	0.876	0.385	0.852	0.823	0.675

POD, postoperative delirium; ROC, receiver operating characteristic curve; AUC, area under the curve; APACHE II, acute physiology and chronic health evaluation II; J, youden index. All continuous variables were analyzed as continuous predictors.

### 3.5 Calibration curve and clinical decision curve analysis

**Figure 5:**
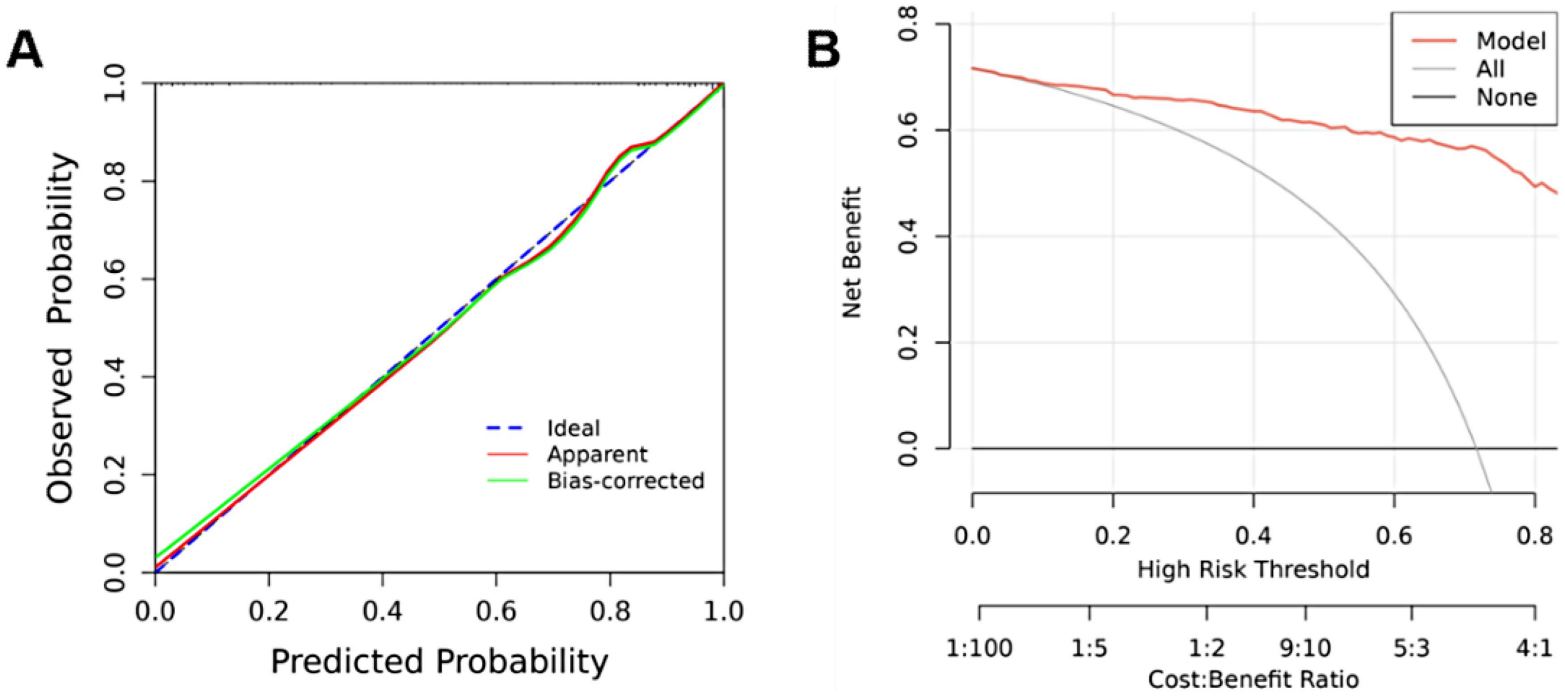
DCA of the combined prediction model for POD. POD, postoperative delirium; DCA, decision curve analysis. **(A)** Calibration curve: The solid red line represents the performance of the combined model; closer alignment with the diagonal line indicates better calibration. **(B)** Decision curve analysis: The *y*-axis represents the net benefit, and the *x*-axis represents the threshold probability for clinical intervention. A model is considered clinically useful if its net benefit exceeds both reference lines across a range of clinically reasonable threshold probabilities.

The calibration curve assessment showed that the five risk factors of Age, Mechanical ventilation duration, Aortic occlusion time, APACHE II score, and Albumin were highly consistent with the actual occurrence probability in the internal validation of the prediction. The average absolute error between the predicted values and the actual values of the two groups was 0.076, as shown in Figure 5A.

The clinical decision curve analysis confirmed that when the probability threshold range was set between 1% and 82%, the net benefit of predicting the occurrence of POD through the five risk factors of Age, Mechanical ventilation duration, Aortic occlusion time, APACHE II score, and Albumin was relatively high. See Figure 5B.

## 4 Discussion

POD is a common severe neurological complication after cardiac surgery, closely related to age, increased medical costs, and long-term decline in cognitive function (26, 27). Although its clinical significance has been widely recognized, the pathogenesis of POD has not been fully elucidated, and early identification of high-risk patients remains challenging (28). This study included patients undergoing cardiac surgery, with a POD incidence rate of 28.15%. Multivariate analysis showed that age, aortic cross-clamping time, mechanical ventilation time, APACHE II score, and albumin level were independent influencing factors for POD. ROC analysis and decision curve further verified the predictive efficacy of the combined model of these indicators. The calibration curve showed that the model had good calibration (average absolute error 0.076), and the clinical decision curve indicated that the net benefit was higher within the probability threshold range of 1% to 82%.

General data comparison is the basis for identifying potential influencing factors of POD. The univariate analysis in this study showed that the incidence of POD was 28.15%, and the age of patients in the POD group was significantly higher than that in the Non-POD group (*P* < 0.05), while there were no significant differences in gender, BMI, educational level and past medical history between the two groups (*P* > 0.05). This result suggests that advanced age is an important demographic risk feature of POD after cardiac surgery, rather than other baseline factors. In elderly patients, the reserve of brain neurons decreases, the function of the neurotransmitter system declines, and the integrity of the blood-brain barrier reduces, significantly weakening their compensatory ability to surgical trauma and anesthesia (29). A meta-analysis in 2023 included 20 studies on cardiac surgery and also found that age was the most stable demographic risk factor for POD (combined OR = 2.50, 95% CI: 1.75–3.56) (30). The susceptibility to POD mainly depends on neurocognitive reserve rather than general physical condition or educational level. The possible reason is that the stress intensity of cardiac surgery far exceeds that of other surgeries, which is sufficient to mask minor differences in cognitive reserve; while age-related neurodegeneration is irreversible and cumulative damage, and its effect is most prominent. This result suggests that age should be used as the primary indicator for risk stratification of POD after cardiac surgery. For elderly patients, even if they are in good general condition, routine delirium prevention strategies should be implemented.

Before delving into the independent risk factors of POD, it is necessary to clarify the clinical significance of each observation indicator and its potential impact on the prognosis of patients. The APACHE II score is a classic tool for assessing the severity and prognosis of critically ill patients. A higher score indicates poorer overall physiological reserve and a greater risk of multiple organ dysfunction. Such patients have a reduced tolerance to surgical and anesthetic stress and are more prone to central nervous system complications (31). The rate of sedative drug use reflects the postoperative analgesic and sedative regimen. Among these drugs, benzodiazepines and others can disrupt sleep patterns and inhibit cholinergic pathways, and have been proven to be independent risk factors for delirium (32). Postoperative hypotension leads to a decrease in cerebral perfusion pressure, causing ischemia and hypoxia in local brain tissues, especially in regions such as the hippocampus and prefrontal cortex that are closely related to cognitive functions. The duration and severity of hypotension directly affect the threshold for neuronal damage (33). The level of albumin not only reflects the nutritional status, but also participates in inflammation regulation as an antioxidant and drug carrier (34). The prolonged duration of aortic occlusion and the extended time of extracorporeal circulation indicate more significant non-physiological blood flow patterns, ischemia-reperfusion injury, and systemic inflammatory response syndrome. A large number of pro-inflammatory factors penetrate the blood-brain barrier, activate microglia, and cause excitotoxic damage to neurons (35). The prolonged duration of mechanical ventilation not only increases the risk of ventilator-associated pneumonia, but also causes sensory deprivation, fragmented sleep and braking-related stress, thereby raising cortisol levels and impairing hippocampus-dependent memory function (36). The above indicators respectively depicted the pathological and physiological network of POD occurrence from three dimensions: overall preoperative condition, intraoperative operation complexity, and postoperative management quality. This study found that the APACHE Ⅱ score, the rate of sedative drug use, the proportion of postoperative hypotension, and the albumin level were lower in the POD group, while the duration of aortic occlusion, the time of extracorporeal circulation, and the time of mechanical ventilation were longer (all *P* < 0.05). These indicators covered the severity of preoperative condition, the intensity of intraoperative operation, and the quality of postoperative management, suggesting that POD is not caused by a single factor but is a concentrated manifestation of multiple pathological and physiological disorders. A 2020 study involving 2,561 CABG patients showed that for every additional hour of mechanical ventilation, the risk of delirium increased by 12% (OR per hour = 1.12, 95% CI: 1.05–1.19), which is consistent with the findings of this study regarding the prolongation of mechanical ventilation time (37). The finding that the albumin level in the POD group was lower was consistent with the result of the multivariate analysis, where albumin was identified as a protective factor (OR = 0.771) (34). Possible causes include the following aspects. Long-term aortic occlusion and cardiopulmonary bypass trigger systemic inflammatory responses, activate microglia and damage the blood-brain barrier; postoperative sedatives interfere with sleep rhythms and neurotransmitter balance; mechanical ventilation prolongation leads to sensory deprivation and braking-related delirium; hypotensive events cause insufficient cerebral perfusion. These factors accumulate and form combined factors that trigger POD. The multiple-dimensional risk factors mentioned above suggest that the prevention of POD requires a comprehensive intervention strategy. It is necessary to prioritize reducing intraoperative operational stress, shortening aortic occlusion and cardiopulmonary bypass times, optimizing sedation regimens, maintaining stable postoperative cerebral perfusion, and implementing early extubation.

Multivariate Logistic regression analysis was used to control confounding factors and identify independent risk factors (38). The results of this study show that older age (OR = 1.134), longer aortic occlusion time (OR = 1.141), longer mechanical ventilation time (OR = 1.372), higher APACHE II score (OR = 1.927), and lower albumin levels (OR = 0.771) are independent influencing factors for POD (*P* < 0.05). Among them, the OR value of the APACHE II score is the largest, suggesting that the severity of the disease has the strongest predictive effect on POD. Additionally, a lower albumin level acts as a protective factor. The discovery of APACHE II as an independent risk factor in 2025's systematic review is consistent. This review indicates that APACHE II is the most commonly included common predictor factor in the ICU delirium prediction model (39). The result that albumin acts as a protective factor is consistent with a prospective study, which found that low albumin levels after surgery are an independent predictor of delirium (34). Studies have shown that APACHE II incorporates multiple acute physiological parameters and reflects the extent of systemic inflammation and metabolic disorders. Delirium, on the other hand, is essentially acute brain failure (40). Albumin has antioxidant, anti-inflammatory and substance transport functions. Albumin has antioxidant, anti-inflammatory and substance transport functions, which can neutralize the elevated oxidative stress products during the perioperative period and protect neurons (41). Among the aforementioned independent risk factors, the APACHE II score can be used to identify high-risk patients before surgery, while the protective effect of albumin suggests that maintaining normal albumin levels during the perioperative period may help reduce the risk of POD. Shortening the time of aortic occlusion and mechanical ventilation is a core controllable intervention target in clinical practice.

ROC analysis is often used to evaluate the ability of each risk factor to distinguish between different disease conditions alone (32). In this study, the AUC values for age, mechanical ventilation time, aortic occlusion time, APACHE II score, and albumin were 0.719, 0.769, 0.677, 0.782, and 0.845 respectively. The AUC of albumin was the highest (0.845), followed by APACHE II score (0.782), suggesting that these two indicators have moderate to above-average predictive efficacy; the SHAP analysis also revealed that the average absolute SHAP value of Albumin was the highest, at 2.81; the AUC of aortic occlusion time was the lowest (0.677), but still had certain predictive value. In terms of sensitivity, age was the highest (0.707), and APACHE II score was the lowest (0.414); in terms of specificity, age was the highest (0.659), while the specificities of mechanical ventilation time, aortic occlusion time, APACHE II score, and albumin were all below 0.2, indicating that the false positive rates of these four indicators were relatively high. This result pattern is similar to that of the 2024 study, which showed that the PRE-DELIRIC model had an AUC of 0.77, a sensitivity of 64.8%, and a specificity of 83.2% in cardiac surgery patients (42). The possible reason for the highest AUC of albumin in this study is that albumin is an integrated indicator reflecting systemic inflammation, nutritional status, and vascular integrity. The early postoperative decrease in albumin can sensitively capture the intensity of the inflammatory cascade reaction; while the AUC of APACHE II score is high but has low sensitivity, possibly because the use of sedative drugs is the result of clinical decision-making, rather than a simple causal exposure. Age has high sensitivity but generally low specificity, indicating that age can be used as a screening indicator to identify most patients with POD, but it will also mislabel many non-POD patients. The AUC of the joint prediction model constructed in this study reached 0.876, with a sensitivity of 85.2% and a specificity of 82.3%, which was superior to each individual indicator. Overall, this suggests that clinical practice requires the construction of a combined model using multiple indicators.

The calibration curve is used to assess the consistency between the predicted probability and the actual occurrence probability, while the clinical decision curve quantifies the net benefits under different probability thresholds (43). The calibration curve of this study shows that the probability of POD occurrence predicted by the combination of five risk factors is highly consistent with the actual observed probability, with an average absolute error of 0.076, indicating that the combined model has good calibration. The decision curve analysis shows that when the probability threshold is within a wide range of 1% to 82%, the prediction model based on these five factors has a high net benefit, indicating that the model has broad clinical applicability. The nomogram model for post-CABG delirium published in 2024 also conducted calibration curve and DCA analyses, and its calibration performance in the training set and test set was good, similar to the results of this study (44). Another study utilized a machine learning ensemble model to predict post-cardiac surgery delirium, with an AUC of 0.887. The calibration curve showed that the predicted probabilities were highly consistent with the actual probabilities. DCA also confirmed its clinical net benefit, further supporting the view that the multi-factor combined model is superior to single-factor prediction (6). The excellent calibration and wide threshold range of the combined model, along with the high net benefits, suggest that this model can effectively guide clinical decisions. The good predictive probability might be due to the fact that the five included variables cover independent risk factors in three stages (preoperative age, intraoperative aortic occlusion time, postoperative mechanical ventilation time, sedative drugs, and albumin), with a complete information dimension; and all are clinical routinely collected indicators, with a low data missing rate and good model robustness.

In addition, in the exclusion criteria of this study, patients who experienced postoperative coma or developed severe complications were excluded. This was mainly based on the following clinical and statistical considerations. Firstly, such patients have a significantly increased risk of delirium due to severe surgical trauma, systemic inflammatory response, and persistent organ dysfunction. The confounding effect is too strong. If they were included, it might mask the predictive value of common and modifiable variables such as age, mild hypoalbuminemia, and moderate hypotension, which is not conducive to constructing an early risk identification model applicable to the majority of patients. Secondly, in a coma or deep sedation state, it is impossible to effectively assess delirium using the ICDSC. The diagnosis is highly uncertain. Forcing their inclusion would instead increase information bias. Therefore, although excluding this group limits the extrapolation of the conclusion to the most critically ill patients to a certain extent, it improves the stability and clinical interpretability of the model in the non-comatose and non-extremely severe complication patient population.

It is particularly worth noting that the multiple independent risk factors identified in this study are modifiable, providing direct targets for clinical prevention strategies. Mechanical ventilation duration (OR = 1.372) can be reduced by daily spontaneous breathing trials combined with sedation interruption, which has been shown to lower delirium risk (45). Low albumin levels (OR = 0.771, 23% risk reduction per 1 g/L increase) are modifiable through perioperative nutritional support and albumin supplementation (46). The APACHE II score (OR = 1.927) reflects underlying organ dysfunction that can be improved via early goal-directed therapy. Aortic cross-clamping time (OR = 1.141) can be minimized by optimizing surgical techniques and myocardial protection strategies. Although age is non-modifiable, it helps identify high-risk populations for targeted prevention. Thus, most significant risk factors identified in this study point to clear clinical intervention targets, supporting a risk-stratified bundled prevention strategy.

However, this study has several limitations. First, it was a single-center study with a relatively small sample size, which limits generalizability; therefore, we plan to validate the five-factor model in an independent prospective multicenter cohort. Second, we did not collect data on preoperative cognitive function or baseline delirium risk, and the observational design may introduce bias due to non-randomized sedative use; thus, future studies will integrate neuroinflammatory biomarkers and develop a dynamic online risk calculator to improve prediction and clinical applicability. In addition, this study excluded patients who experienced postoperative coma or severe complications (Clavien-Dindo grade ≥ IV). Therefore, the research conclusions are mainly applicable to the post-cardiac surgery population that is not in a coma and does not have extremely severe complications. For the most critically ill patients, the predictive efficacy of this model is not yet clear and further targeted research is needed for verification.

In conclusion, this study demonstrates that age, aortic cross-clamping time, mechanical ventilation duration, APACHE II score, and albumin level are independent predictors of POD after cardiac surgery. The combined model shows good discriminative ability (the AUC of albumin reaches 0.845, and the SHAP value is 2.81), excellent calibration (mean absolute error = 0.076), and favorable net benefit across a wide probability threshold range (1%–82%). These findings highlight that POD arises from a multifactorial pathophysiological network spanning preoperative, intraoperative, and postoperative phases. The simplicity and accessibility of the included variables make the model readily applicable in clinical practice. Future multicenter prospective studies are needed to validate and refine the model, ultimately improving perioperative cognitive outcomes in cardiac surgical patients.

## Data Availability

The raw data supporting the conclusions of this article will be made available by the authors, without undue reservation.
